# An Atypical Presentation of Aggressive Oral Pyogenic Granuloma With Oroantral Communication and Bone Erosion

**DOI:** 10.7759/cureus.93237

**Published:** 2025-09-25

**Authors:** Chirag Gupta, Nuzla Agha, Sohini Chakraborty, Tabinda Khan, Aparna Mishra

**Affiliations:** 1 Department of Periodontics, Kothiwal Dental College and Research Centre, Moradabad, IND; 2 Department of Oral Medicine and Radiology, Kothiwal Dental College and Research Centre, Moradabad, IND

**Keywords:** bone, erosion, maxillary sinus, oroantral communication, pyogenic granuloma

## Abstract

Oral pyogenic granuloma is a benign, reactive vascular lesion that commonly affects the oral mucosa and is often triggered by trauma or irritation, presenting as a friable, bleeding mass. A 30-year-old man presented with a recurrent, rapidly growing oral mass in the right maxillary posterior region, causing difficulty in chewing and spontaneous bleeding for one year. Two years subsequent to the extraction of the right maxillary second premolar, a lesion manifested and was subsequently excised through surgical means. One year ago, the lesion re-emerged and progressively expanded to encompass both the alveolar ridge and the buccal vestibule. Clinical examination revealed a 10×12×15 mm erythematous, pedunculated, exophytic growth, which was soft and bleeding on palpation. Cone-beam computed tomography revealed alveolar bone erosion near the maxillary sinus floor. Electrosurgical excision was performed under local anesthesia with the extraction of an adjacent tooth due to lesion infiltration. Intraoperative oroantral communication was identified and closed using a hemostatic agent and sutures. Histopathology confirmed oral pyogenic granuloma, characterized by vascular proliferation and inflammatory infiltrates. Postoperative care included antibiotics, nasal decongestants, and strict oral hygiene, which resulted in uneventful healing by day 10 and no recurrence at nine months. This rare case of aggressive oral pyogenic granuloma with bone destruction and sinus involvement highlights the importance of early diagnosis, thorough excision, and removal of etiological factors, such as trauma, to prevent recurrence. This emphasizes the need for clinicians to consider such atypical presentations in differentials for vascular oral lesions and ensure multidisciplinary management to optimize outcomes.

## Introduction

Oral pyogenic granuloma (PG) is a benign, reactive, vascular lesion commonly encountered in the oral cavity that often arises due to chronic irritation, trauma, or hormonal influences [1]. It is also known as lobular capillary hemangioma, pyogenic granuloma, or telangiectatic granuloma. Characterized by rapid growth, a friable surface, and a tendency to bleed, PG typically presents as a soft erythematous nodule [2,3]. In a comprehensive examination of 244 cases of gingival lesions within the South Indian demographic, Shamim et al. [4] determined that non-neoplastic lesions constituted 75.5% of the cases, with oral pyogenic granuloma emerging as the predominant lesion, representing 52.71% of the cases.

It is typically a benign reactive vascular lesion that is not inherently aggressive [5]. In most cases, PG is non-invasive and does not cause significant tissue destruction or metastasis, distinguishing it from malignant lesions [1]. Histologically, PG shows proliferation of endothelial cells, inflammatory infiltrates, and granulation tissue, which can sometimes mimic malignant conditions such as squamous cell carcinoma, necessitating careful diagnosis through biopsy and imaging [1,4,6]. Although aggressive cases are rare, they highlight the importance of early recognition and management to prevent complications [7].

Although it is frequently observed on the gingiva, its aggressive behavior leading to significant complications, such as oroantral communication (OAC), is exceedingly rare. An OAC, an abnormal connection between the oral cavity and maxillary sinus, is more commonly associated with dental extractions, infections, or trauma. This case report describes a rare instance of aggressive oral PG resulting in OAC and highlights its diagnostic challenges and clinical management. The rarity of this presentation underscores the importance of early recognition, accurate diagnosis, and multidisciplinary management to mitigate complications and to ensure optimal outcomes.

This case report aims to contribute to the limited literature on aggressive PG leading to OAC, emphasizing the need for heightened clinical suspicion and comprehensive diagnostic approaches, including imaging and histopathology, to differentiate it from malignant entities. By detailing the clinical presentation, diagnostic process, and therapeutic interventions, this case highlights the complexities of managing such rare complications and provides insights for clinicians encountering similar presentations.

## Case presentation

A 30-year-old male patient presented to the Department of Periodontology, Kothiwal Dental College and Research Centre, Moradabad, India, complaining of persistent growth in the right maxillary posterior region for one year, causing difficulty in chewing and spontaneous bleeding. He reported no relevant medical history, was a non-smoker, denied alcohol or drug use, and had no family history of similar lesions or malignancies. The growth was first noticed two years before, following the extraction of the upper right second premolar (tooth #15, FDI (World Dental Federation) notation). Initially small, it was excised but recurred within months, growing slowly, reaching its current size. The patient reported occasional nasal regurgitation of fluids, suggesting possible maxillary sinus involvement.

**Figure 1:**
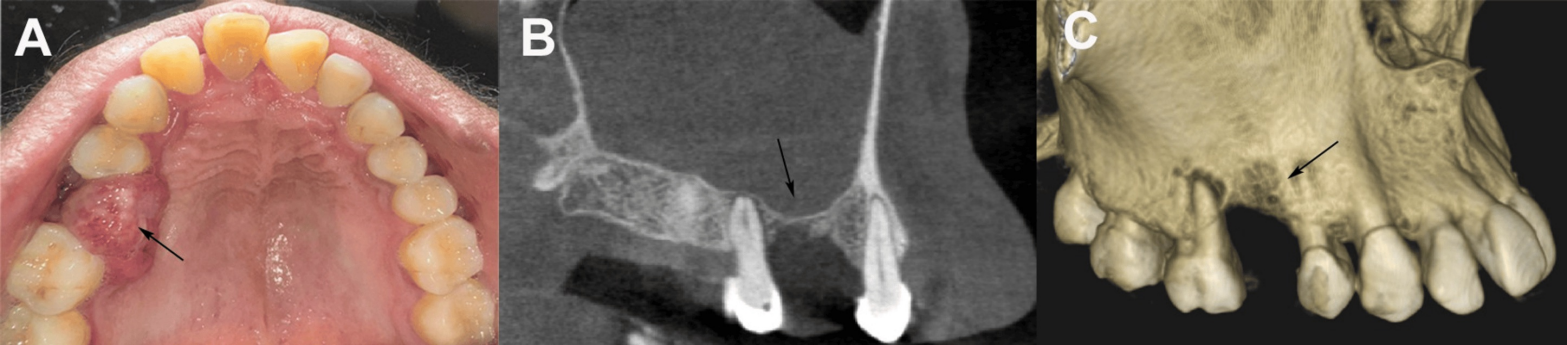
(A) Clinical photograph showing a 10×12×15 mm irregular, soft, pedunculated, exophytic, dome-shaped growth on the edentulous #15 alveolar ridge, with a red, inflamed, lobulated surface and erythematous surrounding mucosa. (B) Cone-beam computed tomography (CBCT) scan revealing erosion of the alveolar bone in the #15 region, approaching the maxillary sinus floor. (C) Additional CBCT view highlighting significant alveolar bone loss in the #15 region, raising concerns for potential oroantral communication. Patient images were obtained with informed consent for publication.

Extraoral examination revealed mild swelling of the right cheek, approximately 1.5 cm in diameter, which was soft and non-tender, with no skin changes or palpable lymph nodes. Intraorally, a 10×12×15 mm irregular, soft, pedunculated, exophytic, dome-shaped growth was observed on the edentulous #15 alveolar ridge, extending from the buccal vestibule to the alveolar ridge and posteriorly along the palatal aspect of the first molar (tooth #16). The surface of the lesion was red, inflamed, and lobulated, with erythematous surrounding mucosa. Palpation revealed a soft, slightly fluctuant, non-reducible, non-compressible, non-tender mass that bled profusely on deep probing, indicating high vascularity. The adjacent teeth were stable, with mild periodontal attachment loss around the upper right first molar (tooth #16, FDI notation) (Figure 1A).

The cone-beam computed tomography (CBCT) scan revealed a soft tissue mass, measuring approximately 10×12×15 mm, eroding the alveolar bone in the #15 region and extending to within 1 mm of the maxillary sinus floor, indicating significant bone destruction and potential OAC (Figure 1B, 1C). Preoperative blood tests, including a complete blood count, coagulation profile, and random blood sugar, were normal, ruling out anemia, coagulopathy, or undiagnosed diabetes. The provisional diagnosis, based on recurrent, vascular, exophytic nature, and bone erosion, was aggressive oral PG, with differentials including peripheral giant cell granuloma, fibroma, hemangioma, or malignancy such as squamous cell carcinoma, although the latter was unlikely given the patient’s profile.

**Figure 2:**
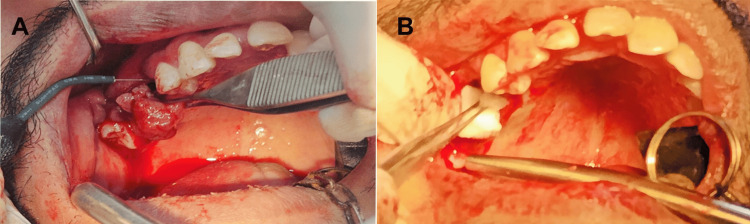
(A) Electrosurgical excision of the lesion. (B) ABgel® gelatin-based hemostatic sponge placement for oroantral communication (OAC) closure. Patient images were obtained with written informed consent for publication.

Surgical excision was performed under local anesthesia (2% lidocaine with 1:100,000 epinephrine) using the electrosurgical unit (ART Electrosurgicals, Taipei, Taiwan), which employed 1.5-1.7 MHz radiofrequency energy at a power supply of 220 V and a current of 0.9 A for precise cutting and coagulation. A surgical excision was performed with a loop electrode in cut-coagulate mode to control bleeding, given the vascularity of the lesion (Figure 2A). Tooth #16 was extracted due to lesion infiltration into the periodontal ligament. Upon identification of the oro-antral communication (OAC) during curettage of the extraction site, the defect, measuring approximately 5 mm in diameter, was meticulously assessed to ensure no active sinus infection or foreign bodies were present. A piece of ABgel® gelatin-based hemostatic sponge (Sri Vishnu Pharma, Hyderabad, India), sized to slightly exceed the communication's dimensions, was gently packed into the defect to achieve hemostasis, promote clot stabilization, and serve as a scaffold for initial tissue regeneration, preventing displacement into the maxillary sinus. The surrounding mucosal edges were debrided to promote healthy granulation and tissue formation. Subsequently, a tension-free primary closure was performed using a buccal advancement flap mobilized via two vertical releasing incisions to ensure adequate mobility without compromising vascular supply. The flap was advanced over the hemostatic agent to cover the defect completely, and sutured in place with interrupted 4-0 Vicryl® resorbable sutures (Ethicon Inc., Johnson & Johnson, New Jersey, USA), incorporating horizontal mattress stitches for secure approximation of the buccal and palatal mucosa. This approach facilitated uneventful mucosal healing and OAC closure, as confirmed on follow-up (Figure 2B). The wound was sutured using 4-0 Vicryl® resorbable sutures. Postoperative instructions included avoiding forceful nose blowing, straw use, smoking, strenuous activity, maintaining oral hygiene with warm saline rinses, and following a soft diet for 24 h. Antibiotics (amoxicillin 500 mg thrice daily for five days) and nasal decongestants (xylometazoline 0.1% nasal drops) were prescribed to prevent infection and sinus complications.

**Figure 3:**
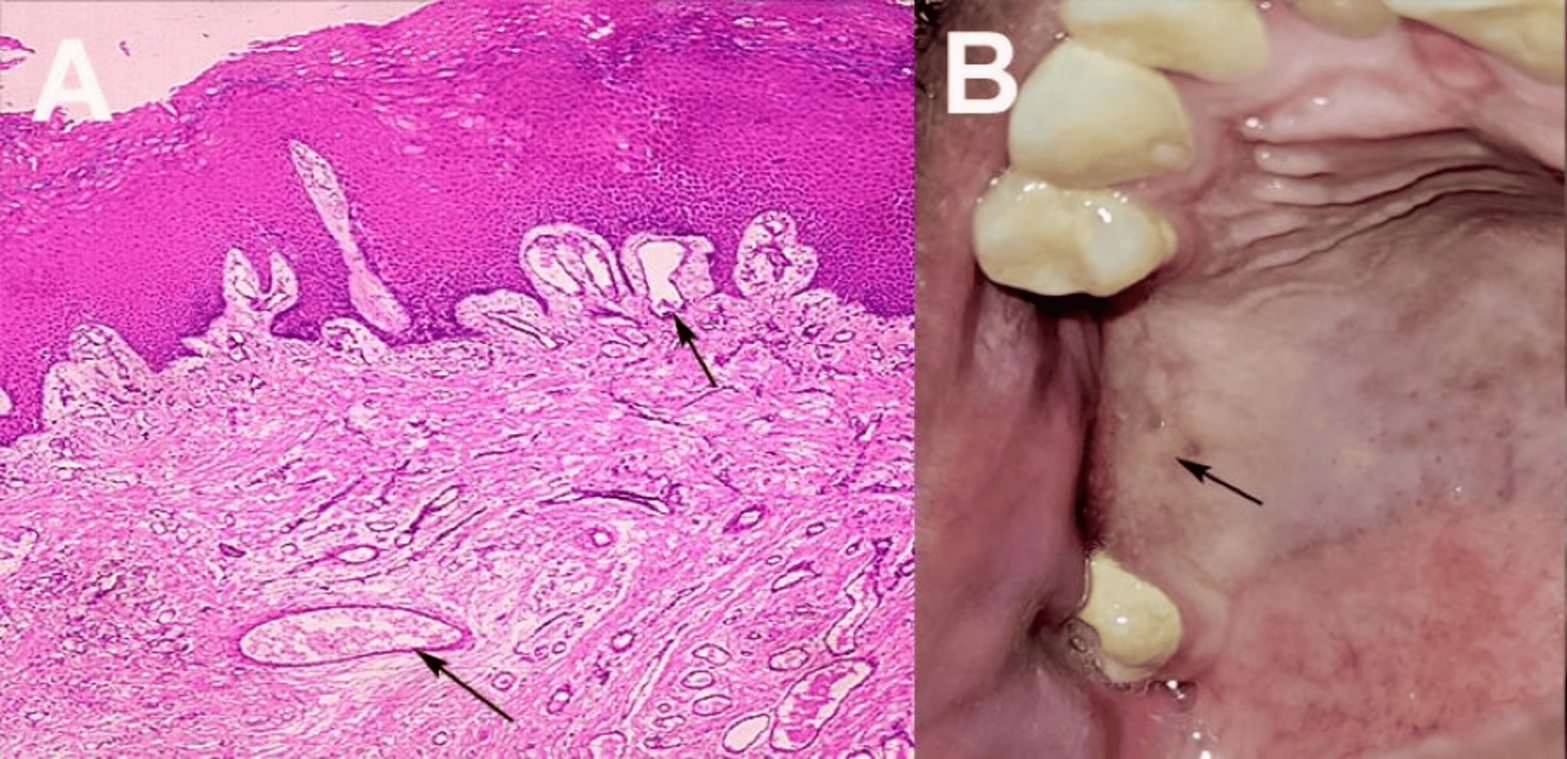
(A) Histopathological image (hematoxylin and eosin stained, viewed at 40x magnification) showing clot formation, subepithelial edema, and numerous proliferated capillaries under the epithelium, characteristic of telangiectatic granuloma (oral pyogenic granuloma). (B) Clinical photograph at nine-month follow-up, demonstrating a fully healed surgical site with no signs of inflammation or recurrence following excision and oroantral communication closure. Patient images were obtained with written informed consent for publication.

Histopathological examination revealed clot formation, subepithelial edema, and numerous proliferated capillaries under the epithelium, consistent with a telangiectatic granuloma (PG), confirming the diagnosis (Figure 3A). On the 10th day of follow-up, healing was uneventful with complete OAC closure. At nine months, the surgical site showed no signs of inflammation or recurrence (Figure 3B). The patient was scheduled for six-monthly recall visits to monitor for recurrence, given the history of the lesion.

## Discussion

PG is a benign, reactive vascular proliferation rather than a true neoplasm, often triggered by local irritation, trauma, or hormonal influences, manifesting as a friable, exophytic lesion prone to bleeding [1]. Although relatively uncommon, it predominantly affects the oral mucosa, with the gingiva being the most frequent site, showing a predilection for the maxilla over the mandible and anterior regions over posterior ones [5,6].

Etiologically, chronic low-grade irritation or trauma, as seen after dental extraction in the present case, plays a key role, alongside hormonal factors favoring young females in the second decade and certain drugs such as cyclosporine, as evidenced by Lee et al.'s [8] three cases in bone marrow transplant patients. In our case, a 30-year-old male patient presented with a history of tooth #15 extraction two years prior, which likely initiated the lesion, with incomplete prior excision contributing to recurrence and aggressive progression, deviating from the typical female predominance.

Clinically, PG presents as a smooth or lobulated, sessile, or pedunculated mass with an ulcerated, vascular surface, often painless but bleeding easily [2], matching the current case's 10×10×10 mm dome-shaped growth in the right maxillary posterior region, extending buccally and palatally, with spontaneous hemorrhage and mastication interference. Masticatory forces might have led to further trauma and progressive growth. Radiographically, PG usually spares the bone, but rare aggressive variants cause superficial erosion or extensive loss. Martins-Filho et al. [9] described an aggressive pregnancy-associated PG mimicking malignancy due to severe bone destruction, while Saravana's [10] review of 137 cases noted four cases with significant bone loss and tooth mobility. Shetty et al. [11] reported a case of aggressive PG of the anterior maxilla in an 18-year-old female patient, leading to severe bone destruction. Thada et al. [12] reported a large PG of the posterior mandible of size 2.5 cm diameter, in a 20-year-old female patient, eroding the upper border of the mandibular canal with a sunray appearance mimicking a malignant tumor.

The present CBCT findings of alveolar erosion near the maxillary sinus floor underscore this aggressiveness, culminating in intraoperative OAC, a complication that is rarely linked to PG. To the best of our knowledge, only two cases of extensive sinus invasion have been reported in the literature [13,14]. The first case was reported in a 13-year-old female patient with aggressive PG of the left posterior maxilla with OAC, which was treated via excision and flap closure [13]. The second case was reported in a 49-year-old female patient with aggressive PG with maxillary sinus involvement and severe maxillary bone resorption [14]. In our case, the male patient, non-smoker status, and absence of systemic factors such as hormones or drugs emphasized trauma as the primary driver, with recurrence possibly from etiological persistence.

In cases of PG, minor trauma, such as post-dental extraction, disrupts the mucosal barrier, creating a portal for nonspecific microorganisms or inflammatory mediators to enter the connective tissue. This initiates a hyperplastic inflammatory process, characterized by endothelial cell proliferation, neovascularization, and granulation tissue formation, leading to a characteristic friable bleeding mass. The growth of the lesion is fueled by persistent low-grade irritation or repetitive trauma, which sustains angiogenic and fibroblastic activity, often resulting in rapid enlargement if untreated [3,13].

Management emphasizes excisional biopsy of the periosteum for definitive diagnosis and treatment [5]. In this instance, electrosurgical excision addressed vascularity with tooth #16 extraction due to periodontal infiltration and OAC closure via ABgel®. Alternative modalities include diode and mid-infrared lasers, cryosurgery, ethanol injection, and corticosteroids, but surgical excision remains the gold standard for aggressive lesions [5]. Despite the fact that PG does not exhibit characteristics indicative of infiltrative behavior or malignant transformation, the incidence of recurrence following straightforward excision is relatively increased (approximately 15.8%), necessitating subsequent re-excision of the lesion in the foreseeable future [15,16]. In the present case report, the patient had a recurrence of PG, leading to aggressive behavior. After retreatment with surgical excision followed by postoperative antibiotics, decongestants and hygiene instructions prevented complications, yielding uneventful healing at 10 days and no recurrence at nine months, with six monthly recalls.

This rare presentation of aggressive PG with OAC highlights diagnostic challenges, mimics malignancy, and underscores multidisciplinary approaches for optimal outcomes, contributing to the sparse literature on sinus-involving variants. The clinical implications of this case include heightened awareness among clinicians that PG, typically benign, can exhibit aggressive behavior with alveolar bone erosion, OAC, and tooth involvement, necessitating prompt biopsy, wide excision, and multidisciplinary management to prevent recurrence and complications, such as sinusitis. This underscores the importance of addressing etiological factors such as post-extraction trauma and considering PG in differentials for vascular oral lesions mimicking malignancy, potentially improving the diagnostic accuracy and patient outcomes in similar rare presentations. Limitations encompass the inherent constraints of a single case report, such as limited generalizability to broader populations, absence of comparative controls or molecular analysis for deeper etiopathogenesis insights, and potential selection bias in reporting only the aggressive variant.

## Conclusions

This case highlights the rare aggressive behavior of PG, typically a benign lesion, leading to alveolar bone erosion, OAC and tooth involvement in a 30-year-old man following post-extraction trauma. Prompt diagnosis via clinical examination, CBCT, and histopathology, coupled with wide electrosurgical excision, tooth extraction, and OAC closure, resulted in uneventful healing and no recurrence at nine months. This underscores the need for heightened clinical suspicion, thorough etiological management, and multidisciplinary approaches to prevent complications in such atypical presentations, contributing valuable insights into the sparse literature on PG with sinus involvement.
